# Smart-phone and medical app use amongst Irish medical students: a survey of use and attitudes

**DOI:** 10.1186/1753-6561-9-S1-A26

**Published:** 2015-01-14

**Authors:** G Browne, D O’Reilly, C Waters, O Tummon, D Devitt, B Stewart, P O’Connor

**Affiliations:** 1 Department of Medicine, National University of Ireland, Western Road, Galway, Ireland

## Background

Studies in the UK and Canada reveal high smart-phone ownership rates with the majority of students viewing these devices as very useful with regards to their clinical education. Worryingly, low awareness basic privacy and security measures appears common amongst medical students. In Ireland, little is known regarding smart-phone app ownership and use. This study sampled Irish undergraduate medical students at a single site.

## Methods

A 31-item questionnaire was developed by the primary researcher following a preliminary literature review and subsequently underwent peer review. The questionnaire was distributed by means of a paper survey. Non-probability convenience sampling was conducted at educational sessions at a single site to students of all years of a medical undergraduate curriculum as per ethics approval. Collected data was analysed using SPSS Statistics 20. The internal consistency of the questionnaire as measured by Cronbach’s alpha was high (α=0.951).

## Results

The survey response rate was 34.8% (317/909) with 80.8% (256/317) of respondents owning a smart-phone. A greater percentage of preclinical students, 83.4% (151/181) owned smart-phones as compared to older students, of which 77.3% owned such a device (105/29). More clinical students (78.1%) used medical apps as compared to preclinical students (57%). The two most popular brands were Apple and Samsung devices. Of those who owned a smart-phone, 65.6% (168/ 256) reported using medically-related apps. Students used apps predominately to aid their study. While 69.9% (179/256) of respondents trusted the information provided by the medical apps they used, only 42.2% (108/256) verified whether app content was correct. In relation to other learning methods, 38.3% (98/256) said they would prefer to use an app instead of a textbook, 23% (59/256) as compared to a lecture, although 50.8% (130/256) would prefer an app to other online information.

## Conclusions

High rates of smart-phone ownership and medical app use exist amongst Irish medical students. While the majority of students trust the apps they use, only 42% verified whether the content of the apps they used was correct. Students require greater guidance when using apps as part of their learning. Universities should educate students regarding such use and provide them with recommendations and guidelines of app use as approved by faculty following a peer review process.

